# *In vivo* Analysis of the Resistance of the Meshes to *Escherichia coli* Infection

**DOI:** 10.3389/fsurg.2021.644227

**Published:** 2021-06-24

**Authors:** Xinsen Xu, Ming Zhan, Xinxing Li, Tao Chen, Linhua Yang

**Affiliations:** ^1^Department of Biliary-Pancreatic Surgery, School of Medicine, Renji Hospital, Shanghai Jiao Tong University, Shanghai, China; ^2^Department of General Surgery, Changzheng Hospital, The Second Military Medical University, Shanghai, China

**Keywords:** resistance, *Escherichia coli*, infection, synthetic mesh, biologic mesh, hernia

## Abstract

**Background:** The mesh infection is mostly related to the gram-negative bacteria, such as Escherichia coli (*E. coli*) for emergency surgery of incarcerated hernia. However, few study investigated the effects of *E. coli* concentration, mesh materials and antibiotic prophylaxis on mesh infection after hernioplasty. The aim of this study was to evaluate the bacterial resistance to *E. coli* for three different materials of mesh, and to measure the minimum *E. coli* concentration for mesh infection with and without antibiotic prophylaxis in a rat model.

**Methods:** Three types of mesh (polytetrafluoroethylene, polypropylene, and biologic meshes) were used in the repair of an acute ventral hernia rat model in the setting of different concentrations of *E. coli* loads and antibiotics. At the 8th day after surgery, mesh samples were sent for microbiologic and histologic analyses.

**Results:** The positive rates of bacterial culture increased with *E. coli* concentration. The biologic mesh showed better bacterial resistance compared to polytetrafluoroethylene mesh and polypropylene mesh when the concentration of *E. coli* ranges from 10^6^ CFU/ml to 10^8^ CFU/ml (*P* = 0.002 and *P* = 0.029, respectively). Prophylactical ceftriaxone treatment could not decrease the colonization rate of *E. coli* at 10^6^ CFU/ml or 10^8^ CFU/ml in each group (*P* > 0.05). The scores of neovascularization in polypropylene mesh and biologic mesh were similar, which was higher than that of polytetrafluoroethylene mesh (*P* < 0.05). Compared with other meshes, biologic mesh showed better tolerance to 10^6^ CFU/ml *E. coli* with respect to inflammation, depth of inflammation, neovascularization, cellular repopulation and foreign body giant cells.

**Conclusion:** The biologic mesh had better *E. coli* resistance compared to polytetrafluoroethylene mesh and polypropylene mesh when the *E. coli* concentration is higher than 10^6^ CFU/ml in rats. Antibiotic prophylaxis was useful when the contamination was not particularly severe.

## Introduction

New complications have arisen since hernioplasty became the main surgical approach for hernia repair, such as seroma, chronic pain, mesh infection and migration (1, 2). For incarcerated or strangulated hernia with or without bowel resection, the infection rate is even higher because of potential contamination, which is reported to be 1.0–7.4%, compared with 0.5–1.0% for elective operations (3–5). Biomaterial infection is still one of the serious complications which may require a second surgery to remove the implants. Recently, anti-infective biomaterials were used to prevent infections that were correlated with medical device, with strategies such as bacteria repelling and antiadhesive surfaces, antibacterial coatings, nanostructured materials, and so on (6).

Many studies have advocated the use of biological mesh under potential contaminated conditions. However, the infection rate is still high even using the biologic mesh (7). In a 5-year period retrospective study, Rosen et al. reported that the wound complication rate was 47.7% in the repairs of abdominal wall defects using biological mesh under infected condition, of which 45.9% wound infections required a second operation (8). By searching multiple electronic databases, Huerta et al. concluded that there was insufficient evidence to determine the clinical efficacy of biological mesh for the repair of abdominal wall hernia (9). On the other hand, Majumder et al. reported that using synthetic sublay mesh might lead to a significantly lower wound morbidity (10). Perez-Kohler et al. also found that the conventional non-absorbable polymer materials might be better candidates for contaminated surgical field because of the low bacterial loads (11). Thus, the best choice of mesh materials in contaminated situation is still controversial. The previous evidence is limited in quantity and quality. It does not support the advantage of biologic over synthetic meshes under infected condition. Kockerling et al. summarized that biologic meshes could not be recommended for routine use in elective hernia repair. They should only be considered for contaminated surgical fields (12).

The biomaterial-associated infection is associated with different bacterial species, concentrations and adherence property. Each bacterial species may have specific adherence properties for prosthetic graft (13). While the most common bacteria presented in mesh infection of elective operation is gram-positive, such as *Staphylococcus aureus* or *Staphylococcus epidermidis* which accounts for 90% of mesh-related infection, our previous study found that for emergency surgery of incarcerated hernia, the mesh infection is more related to the gram-negative bacteria, such as Escherichia coli (*E. coli*) (14, 15). *E. coli* is the common bacterial species which colonize the gastrointestinal tract. For an incarcerated or strangulated hernia repair, the risk of *E. coli* infection increases. As mentioned by the WSES guidelines, in contaminated hernia repair, the most frequently observed pathogen was the *E. coli* (16). Yang et al. demonstrated that the strains of bacteria cultured from would drainage were the same as those from fluid in hernia sac (14). Thus, the mechanism of *E. coli* infection might be the gut bacterial translocation. In immunosuppressed patients, the adherence of *E. coli* to the prosthesis might even trigger sepsis (17, 18). However, there are few studies comparing the resistance to *E. coli* between biologic and synthetic meshes.

According to the Americas Hernia Society, there are mainly two kinds of mesh for hernia repair, namely, the biomaterial and biological mesh. The biomaterial mesh might be either permanent or absorbable. As suggested by the Society of American Gastrointestinal and Endoscopic Surgeons, absorbable mesh did not show significant advantages over permanent mesh, as it is correlated with a higher rate of fistula formation. Thus, the absorbable mesh was not included in this study. Permanent biomaterial mesh usually included polypropylene, polytetrafluoroethylene (PTFE) and polyester mesh. The polyester mesh was reported to be associated with higher infection, intestinal obstruction, hernia recurrence and fistula rates (19). Thus, it was seldom used currently and was excluded from the study. Consequently, three kinds of mesh materials, namely, the polytetrafluoroethylene mesh, polypropylene mesh and biological mesh, were selected as representative meshes in this study. Polypropylene is probably the most extensively used mesh for hernia repair. It is associated with vigorous inflammatory response, ensuring its durability. However, the inflammation might also lead to the tissue adhesion (19). Polytetrafluoroethylene is a microporous woven mesh that usually has two sides - one smooth side with small pores to reduce intestinal adhesions and one side of larger pores with ridges and groves for tissue ingrowth (20). Biological mesh is derived from human, bovine and porcine tissue that is decellularized to a remaining collagen matrix to support remodeling and new collagen deposition (19). Thus, the first objective of the study was to evaluate the bacterial resistance to *E. coli* for different materials of mesh.

On the other hand, studies have shown that the presence of an implanted material will notably reduce the *S. aureus* concentration to only 10^2^ CFU that would be sufficient to trigger an implant infection (7, 21, 22). However, few study investigated the effects of *E. coli* concentration, mesh materials and antibiotic prophylaxis on mesh infection after hernioplasty. Thus, the second aim of this study was to measure the minimum *E. coli* concentration for mesh infection with and without antibiotic prophylaxis in a rat model.

## Materials and Methods

### Animals and Ethics Statement

This study was approved by the Ethical Committee of Renji hospital, Shanghai Jiao Tong University School of Medicine. Two hundred and ten male Wistar rats (range from 240 to 270 g in weight) were used in this study. The protocols for the use of animals were approved by the Department of Laboratory Animal Sciences, Shanghai Jiao Tong University School of Medicine. All animals were obtained from department of animal science (School of Medicine, Shanghai Jiao Tong University) and housed in specific pathogen-free (SPF) rooms with temperature of 20–23°C. Water and pads were sterilized and refreshed daily. All animals were treated in compliance with the guide for the care and use of Laboratory Animals (NIH Publication No. 8023, revised 1978).

### Bacterial Inoculum Preparation

*E. coli* (ATCC #25922) bacterial strains were obtained from microbiology laboratory, Renji Hospital, School of Medicine, Shanghai Jiao Tong University. An aliquot of *E. coli* was cultured in a 2 ml Luria Bertani broth (LB) for 24 h with a temperature of 37°C. The cultures were washed in saline. Spectrophotometry (OD600) was utilized to detect the culture concentration. Each culture was brought to the desired concentration (10^2^ CFU/ml, 10^4^ CFU/ml, 10^6^ CFU/ml and 10^8^ CFU/ml) in 0.9% sterile saline and verified by plating serial ten-fold dilutions of the final solutions used during surgery.

### Mesh Materials and Study Design

Three kinds of mesh materials, namely, the polytetrafluoroethylene mesh, polypropylene mesh and bovine pericardium derivatives, were selected as representative meshes, as they were commonly used in clinical practice in China.

The polytetrafluoroethylene mesh used in this study was the Mycromesh (GORE, USA), which was a multilaminar (multifilament) prosthesis with 2 mm-diameter perforations. However, it was recently discontinued by the manufacturer due to the new mesh products. The knitted monofilament reticular Marlex mesh (Bard, USA) was used as the polypropylene mesh in this study. With respect to the biological mesh, the bovine pericardium derivatives (ThormalGEN Guanhao Biotechnology, Co., Ltd., China) was utilized. Meshes were cut into uniform squares (2.5 × 2.5 cm) when surgeries were performed.

Wistar rats were randomly grouped according to the concentration of bacteria and antibiotics as: Group A (Control group, dropped with 0.9% NaCl), Group B (dropped with *E. coli* of 10^2^ CFU/ml), Group C (dropped with *E. coli* of 10^4^ CFU/ml), Group D (dropped with *E. coli* of 10^6^ CFU/ml), Group E (dropped with *E. coli* of 10^8^ CFU/ml), Group F (dropped with *E. coli* of 10^6^ CFU/ml and injected with ceftriaxone of 30 mg/kg) and Group G (dropped with *E. coli* of 10^8^ CFU/ml and injected with ceftriaxone of 30 mg/kg). Each group was divided into 3 subgroups (*n* = 10 per subgroup): subgroup a (polytetrafluoroethylene mesh), subgroup b (polypropylene mesh) and subgroup c (ThormalGEN mesh).

### Mesh Infection Rat Model

Based on the previous mesh infection rat model from Bellows et al., we developed our own model (23). We chose the abdominal linea alba incision instead of the dorsal skin incision, trying to keep the model more consistent with human hernia repair. Unlike the commonly utilized sublay model or onlay model, we also removed the bilateral abdominal rectus on each side and covered the resulting defect by a mesh that is fixed on the flat abdominal muscles. The peritoneum is left intact below the mesh and the mesh is placed in an onay-bridging position regarding the remaining abdominal wall.

All animals were anesthetized with 10% chloral hydrate (3 ml/kg, intraperitoneal injection). The abdominal skin was clipped and cleaned with povidone iodine. Then the skin was allowed to dry for 2 min. Using sterile techniques, a 2.5 cm longitudinal incision in the linea alba of the rats was made. Then a pocket was developed by removing the bilateral abdominal rectus about 2 × 2 cm, that only the peritoneum was retained. Subsequently, a 2.5 × 2.5 cm patch was placed in the defection, covering the muscles about 0.5 cm. Once the mesh was placed, the bacterial inoculum suspension or saline was pipetted onto the top of the implanted mesh (1 ml). All wounds were closed using absorbable suture (5-0 Ethicon, Johnson & Johnson, USA) 15 min after the bacterial liquid infiltrated the incision. Antibiotic prophylaxis (ceftriaxone 30 mg/kg) was injected intramuscularly 30 min before operation for Group F and Group G.

The rats were housed individually with food and water. The conditions of the incisions were daily observed and recorded, such as hematoma, local infection, sepsis, purulent drainage and wound dehiscence. Wound infection was classified according to the CDC standard (24, 25). Eight days after implantation, all animals were humanely euthanized to harvest the samples for histologic and microbiologic analyses, as described below.

### Collection of Samples

On the 8th day, rats were intraperitoneally injected with a lethal dose of chloral hydrate solution, and a dissection of 3 cm in length was performed 2 cm paralleled to the wound. The mesh pieces and surrounding abdominal tissues of 2.5 × 2.5 cm were excised carefully under sterile conditions, ensuring the whole tissue-prostheses interfaces were included. The implant was divided into two equal pieces, of which one was submerged in 1 ml 0.9% sterile saline and vortexed for bacterial recovery, and the other was fixed for 24 h in 10% buffered formalin for histologic analyses.

### Bacterial Recovery From Explanted Meshes

Bellows et al. have performed several preliminary *in vitro* experiments to determine the most consistently effective method of bacterial recovery (23, 26). They found that vortexing the meshes in sterile saline yielded bacterial recovery rates of 98–99% from two different biologic materials. Thus, in our study, half of explanted mesh was submerged in a tube containing 1 mL of sterile saline and was then vortexed to dissociate adherent bacteria, as described previously (23, 26, 27). Then the serial ten-fold dilutions were plated in triplicate on LB agar and incubated at 37°C for 16–24 h prior to counting colonies. Meshes were scored as positive if the inoculated strain was detected from the cultured sample.

### Histologic Analysis

Samples were fixed in 10% buffered formalin for 24 h and embedded in paraffin. Ten sections, five micrometers thick, were cut from each sample. They were stained for Hematoxylin and Eosin (HE) and Immunohistochemistry (IHC). For HE staining, ten non-overlapping fields in each section were evaluated at 400 × magnification by a pathologist who was blinded to treatment. The evaluation was based on the grading system in Table 1, described by Bellows et al. (23). Characteristics for acute inflammation included presence of polymorphonuclear leukocytes (PMN), depth of the acute inflammatory response, neovascularization, cellular repopulation, and foreign body reaction. For IHC staining, the tissue sections were deparaffinized. They were treated with 3% H_2_O_2_ for 20 min. Samples were autoclaved in 10 mM citric sodium (pH 6.0) for 30 min to retrieve antibody-binding epitopes, washed with PBS and then incubated with CD64 primary antibodies (1:100, Cat. # ab140779, Abcam, USA) 4°C overnight. Then the samples were incubated at room temperature with biotinylated secondary antibody and streptavidin-horseradish peroxidase for 1 h, followed by detection using the DAB system. Histopathologic scores were calculated. Digital images were captured using Olympus IX71 microscope (Olympus Japan, Inc.).

**Table 1:** Histologic scoring system.

**Physiologic variable**	**Assigned score**	**Corresponding host response**
Inflammation	1	0–4 PMNs per HPF
	2	5–20 PMNs per HPF
	3	>20 PMNs per HPF
	4	PMN too numerous to count
Depth of inflammation	1	No inflammatory cells present
	2	Inflammatory cells within 1/3 of mesh framework
	3	Inflammatory cells within 2/3 of mesh framework
	4	Inflammatory cells throughout mesh framework
Neovascularization	1	No capillaries in HPF
	2	1–4 capillaries per HPF
	3	5–10 capillaries per HPF
	4	>10 capillaries per HPF
Cellular repopulation	1	No fibroblast nuclei present
	2	Fibroblast nuclei within 1/3 of mesh framework
	3	Fibroblast nuclei within 2/3 of mesh framework
	4	Fibroblast nuclei throughout mesh framework
Foreign body giant cells	1–10	Absolute number of FBGCs per HPF

*PMN, polymorphonuclear leukocyte; HPF, high-powered field (400 ×); FBGC, foreign body giant cell*.

*This table is an adaptation of the table by Bellows et al. (19)*.

### Statistical Analysis

All data were reported as mean ± standard deviation (SD). Statistical analyses were performed using SPSS 19.0 software with *P* < 0.05 considered significant. Analysis of variance was used to determine the differences among these three groups (polytetrafluoroethylene mesh group, polypropylene mesh group and ThormalGEN mesh group), followed by pairwise multiple comparisons using the Holm-Sidak method to identify specific differences between the groups. Comparisons between two groups were performed using the *t*-test. Correlations between two groups were evaluated using the CORR procedure. Count data was analyzed by CMH-X^2^ test or Fisher exact test.

## Results

### Macroscopic Examination

According to the CDC standard for surgical site infections (US), there were 45 cases identified as surgical wound infections in total, while none was identified in the saline controls (1 in Group Ba, 4 in Group Ca, 1 in Group Cb, 8 in Group Da, 2 in Group Db, 1 in Group Dc, 8 in Group Ea, 5 in Group Eb, 3 in Group Ec, 2 in Group Fa, 1 in Group Fb, 1 in Group Fc, 4 in Group Ga, 2 in Group Gb, and 2 in Group Gc; Table 2). All of the 45 cases were patch infection, of which 13 cases had incision dehiscences while 32 cases had purulent drainage. Five of the 210 animals (2.4%) died of complications of anesthesia, including one case in each of the Group Aa, Ea, Db, Cc, and Dc. The post-operative recovery was normal for the rest rats and none other rats died during the entire experimental period.

**Table 2:** Cases identified as surgical wound infections.

**Inoculum size (*E. coli*)**	**Study group no. colonized/total (%)**			***P*-value**
	**Polytetrafluoroethylene (a)**	**Polypropylene (b)**	**ThormalGEN (c)**	
A Contrast (0.9% NaCl)	0/9 (0)	0/10 (0)	0/10 (0)	–
B (10^2^ CFU/ml of *E. coli*)	1/10 (10)	0/10 (0)	0/10 (0)	1.000
C (10^4^ CFU/ml of *E. coli*)	4/10 (40)	1/10 (10)	0/9 (0)	0.093
D (10^6^ CFU/ml of *E. coli*)	8/10 (80)	2/9 (22)	1/9 (11)	0.005
E (10^8^ CFU/ml of *E. coli*)	8/9 (89)	5/10 (50)	3/10 (30)	0.040
F (10^6^ CFU/ml of *E. coli* + ceftriaxone)	2/10 (20)	1/10 (10)	1/10 (10)	1.000
G (10^8^ CFU/ml of *E. coli* + ceftriaxone)	4/10 (40)	2/10 (20)	2/10 (20)	0.668

### Microbiologic Findings

**Figure 1:**
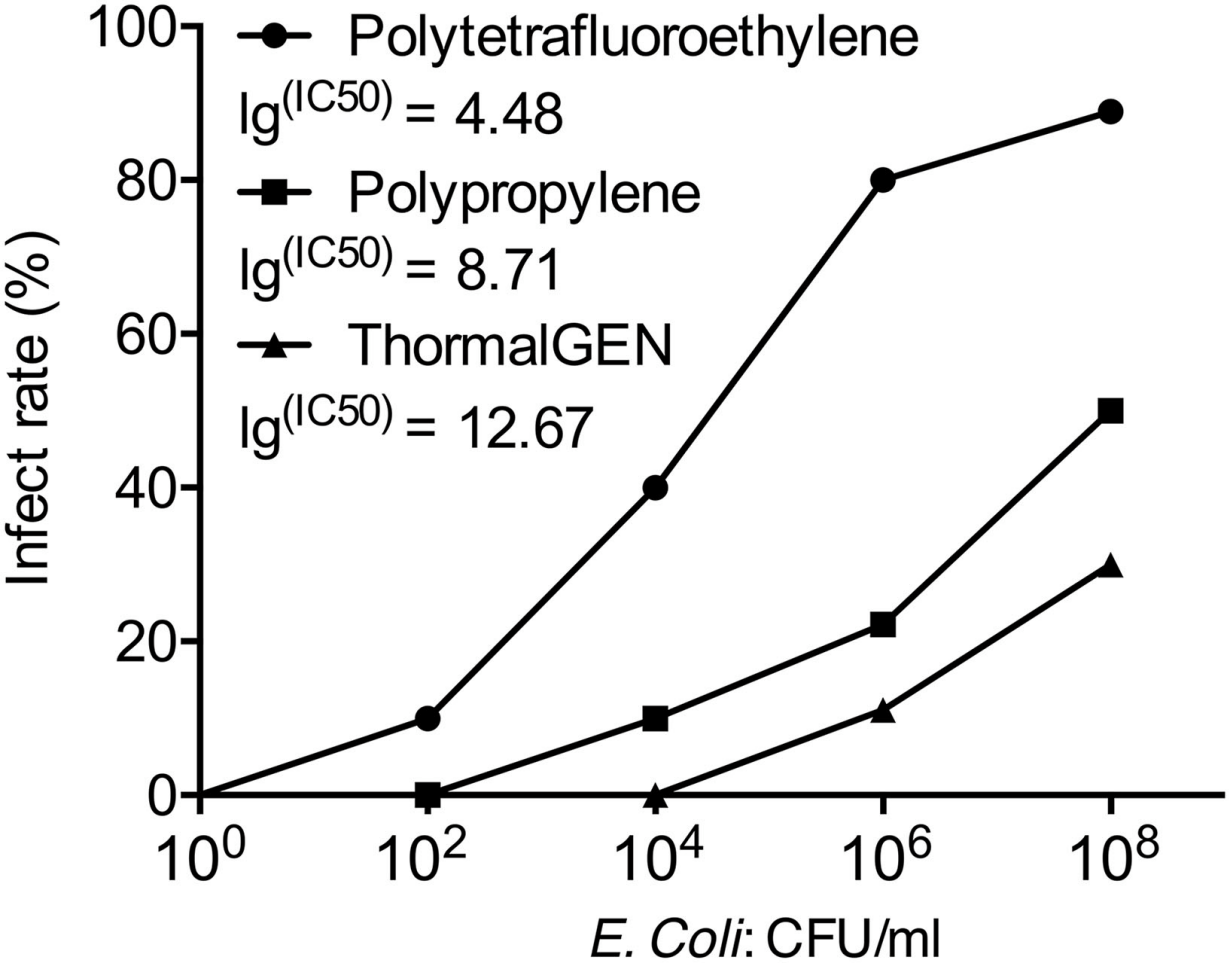
The infection rate of 3 types of mesh with different concentrations of *E. coli*. The IC50 value was presented as the *E. coli*. concentration that infected 50% of rats relative to the untreated control.

**Figure 2:**
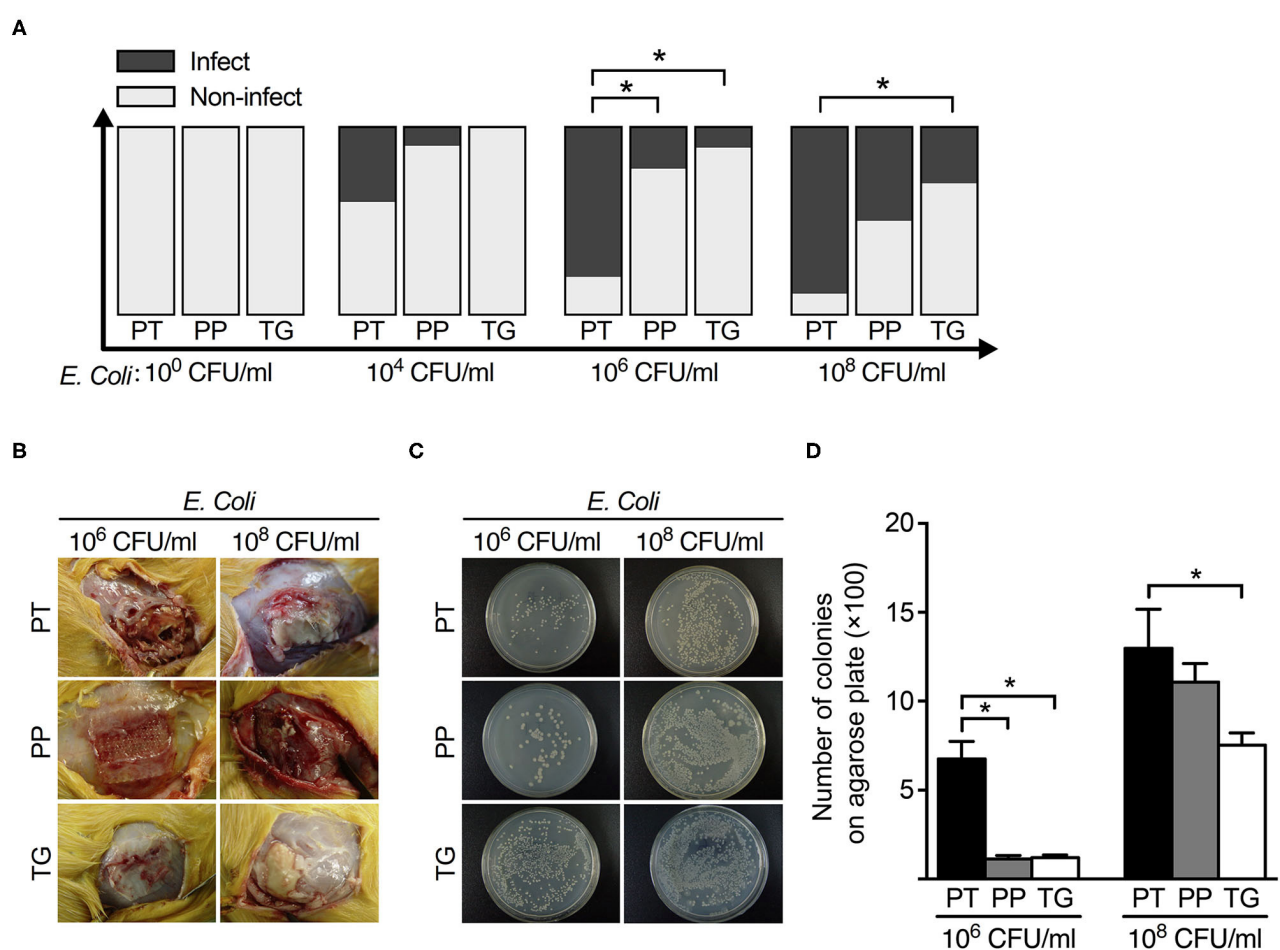
The relationship between mesh types and postoperative infection rate with different concentrations of *E. coli*. **(A)** The histogram of infection rate of 3 types of mesh (polytetrafluoroethylene, polypropylene, and ThormalGEN) with different concentrations of *E. coli*. **(B)** Representative images of postoperative infection with 10^6^ CFU/ml and 10^8^ CFU/ml *E. coli*, respectively. **(C)** Bacterial recovery from the 3 types of mesh after inoculation with 10^6^ CFU/ml and 10^8^ CFU/ml *E. coli*, respectively. **(D)** Analysis of the clone number of *E. coli* in different groups. *P < 0.05, Student's *t*-test. PT, Polytetrafluoroethylene; PP, Polypropylene; TG, ThormalGEN.

Overall, 29% (60 cases of 205) of the explanted meshes revealed the presence of viable *E. coli*. The rates of mesh colonization in different groups were shown in Table 3. There was a positive correlation between the wound infection rate and the positive bacterial culture rate (Figures 1, 2).

**Table 3:** *Escherichia coli* recovery from explanted meshes.

**Inoculum size (*E. coli)***	**Study group no. colonized/total (%)**			***P*-value**
	**Polytetrafluoroethylene (a)**	**Polypropylene (b)**	**ThormalGEN (c)**	
A Contrast (0.9% NaCl)	0/9 (0)	0/10 (0)	0/10 (0)	–
B 10^2^ CFU/ml of *E. coli*	1/10 (10)	0/10 (0)	0/10 (0)	1.000
C (10^4^ CFU/ml of *E. coli*)	4/10 (40)	1/10 (10)	1/9 (11)	0.300
D (10^6^ CFU/ml of *E. coli*)	9/10 (90)	4/9 (44)	1/9 (11)	0.002
E (10^8^ CFU/ml of *E. coli*)	8/9 (89)	7/10 (70)	3/10 (30)	0.029
F (10^6^ CFU/ml of *E. coli* + ceftriaxone)	4/10 (40)	2/10 (20)	1/10 (10)	0.430
G (10^8^ CFU/ml of *E. coli* + ceftriaxone)	8/10 (80)	4/10 (40)	2/10 (20)	0.037

The positive rate of bacterial culture increased with the bacterial concentration. The ThormalGEN mesh has the lowest colonization rate at each *E. coli* concentration, although it was not statistically significant at 10^2^ CFU/ml or 10^4^ CFU/ml (*P* = 1.000 and *P* = 0.300, respectively; Table 3 and Figure 2). At the inoculum of 10^6^ CFU/ml or 10^8^ CFU/ml, the ThormalGEN mesh was more resistant to *E. coli* than polytetrafluoroethylene mesh and polypropylene mesh (*P* = 0.002 and *P* = 0.029, respectively; Table 3 and Figure 2).

On the other hand, injection with ceftriaxone prophylactically did not significantly decrease the colonization rate of *E. coli* at 10^8^ CFU/ml in each group (*P* > 0.05). More specifically, in the polytetrafluoroethylene group, the infection rate was 8/9 without antibiotic prophylaxis, compared with 8/10 with antibiotic prophylaxis (*P* > 0.05). In the polypropylene group, the infection rate was 7/10 without antibiotic prophylaxis, compared with 4/10 with antibiotic prophylaxis (*P* > 0.05). In the ThormalGEN group, the infection rate was 3/10 without antibiotic prophylaxis, compared with 2/10 with antibiotic prophylaxis (*P* > 0.05). Although the infection rate was decreased after prophylactically use of ceftriaxone, it did not reach statistical significance. Likewise, at the inoculum of 10^6^ CFU/ml *E. coli*, the decrease trend of infection rate was more obvious after the use of antibiotics. However, prophylactically use of ceftriaxone was still not able to significantly reduce the colonization rate in polytetrafluoroethylene mesh (9/10 without antibiotics vs. 4/10 with antibiotics; *P* > 0.05), polypropylene mesh (4/9 without antibiotics vs. 2/10 with antibiotics; *P* > 0.05) and ThormalGEN mesh (1/9 without antibiotics vs. 1/10 with antibiotics; *P* > 0.05).

### Histological Findings

**Figure 3:**
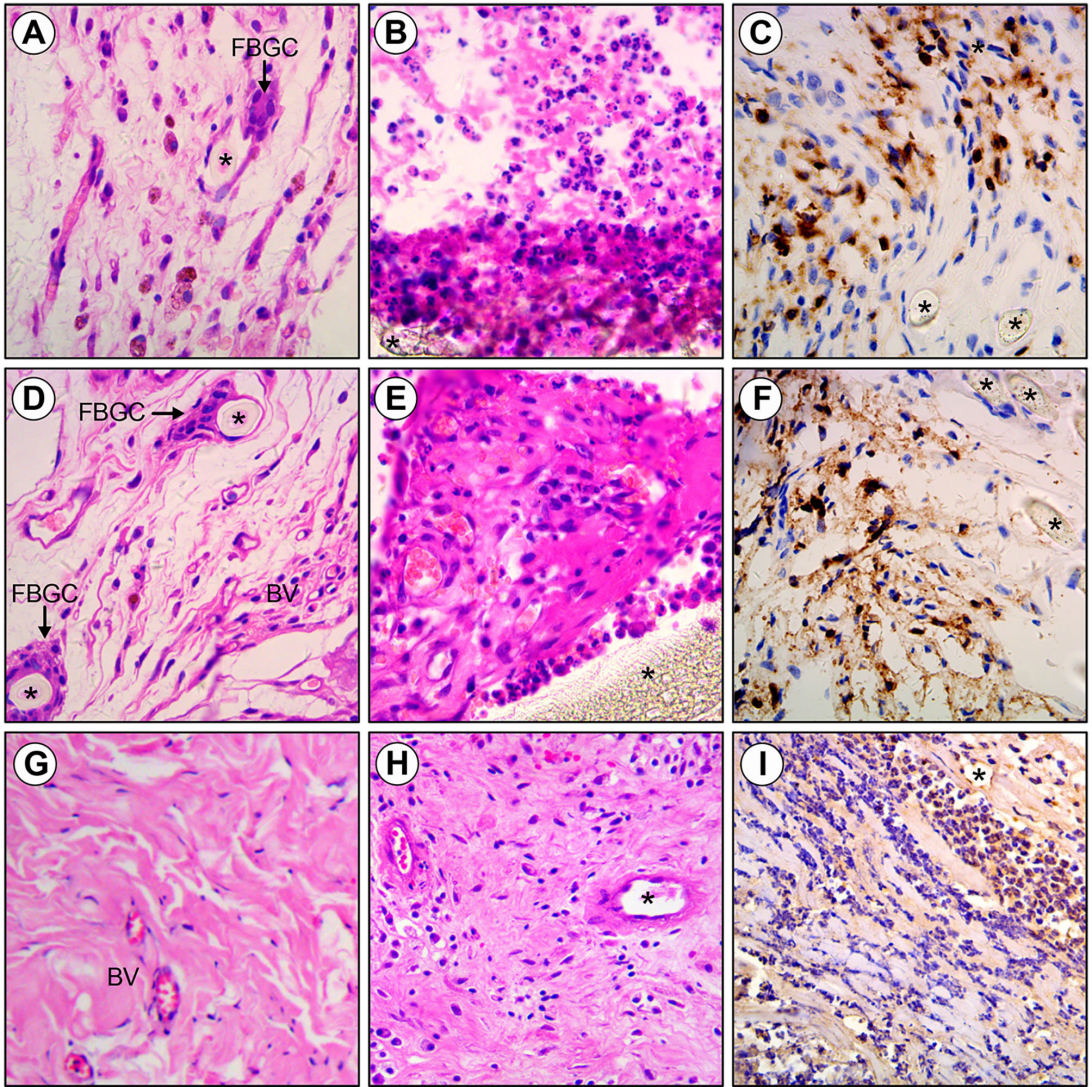
Histology of Polytetrafluoroethylene, Polypropylene and ThormalGEN explants. **(A)** Polytetrafluoroethylene mesh inoculated with saline demonstrating foreign body giant cells (FBGC) and minimal inflammation adjacent to monofilament polyester fibers (asterisk). **(B,C)** Polytetrafluoroethylene inoculated with *E. coli* showing the acute inflammatory cell infiltrate and collagen deposition surrounding intact mesh fibers (asterisk). **(D)** Polypropylene mesh inoculated with saline demonstrating new blood vessel (BV), foreign body giant cells (FBGC), and minimal inflammation adjacent to monofilament polyester fibers (asterisk). **(E,F)** Polypropylene inoculated with *E. coli* showing the acute inflammatory cell infiltrate, collagen deposition, and new blood vessel formation surrounding intact mesh fibers (asterisk). **(G)** ThormalGEN mesh inoculated with saline showing minimal inflammation and new blood vessel (BV) formation. **(H,I)** ThormalGEN mesh inoculated with *E. coli* revealing the presence of moderate inflammation reaction and mesh degradation (asterisk). Detection method: HE and immunohistochemistry staining, bar = 50 μm.

The results of histological findings of group D (10^6^ CFU/ml *E. coli*), group E (10^8^ CFU/ml *E. coli*) and group A (saline) were shown in Table 4 and Figure 3. When dropped with 0.9% NaCl, none of the meshes showed inflammation. However, with respect to depth of inflammation, cellular repopulation and foreign body giant cells, the ThormalGEN mesh outperformed the polytetrafluoroethylene mesh and polypropylene mesh (*P* < 0.05). The scores of neovascularization in polypropylene mesh and ThormalGEN mesh were similar. However, they were higher than that of the polytetrafluoroethylene mesh (*P* < 0.05) (Table 4).

**Table 4:** Mean histologic scores in response to colonization with saline, 10^6^ CFU/ml *E. coli and* 10^8^ CFU/ml *E. coli*.

**Histologic parameter**	**a**			**b**			**c**		
	**polytetrafluoroethylene**			**Polypropylene**			**ThormalGEN**		
	**A Contrast (0.9% NaCl)**	**D** **10^**6**^ CFU/ml** ***E. coli***	**E 10^**8**^ CFU/ml** ***E. coli***	**A** **Contrast (0.9% NaCl)**	**D 10^**6**^ CFU/ml** ***E. coli***	**E** **10^**8**^ CFU/ml** ***E. coli***	**A Contrast (0.9% NaCl)**	**D** **10^**6**^ CFU/ml** ***E. coli***	**E 10^**8**^ CFU/ml** ***E. coli***
Inflammation	1.2 (0.25)	3.2 (0.54)^*^	3.8 (0.33)^*^	1.2 (0.15)	2.4 (0.30)^*^	3.4 (0.43)^*^	1.2 (0.45)	2.0 (0.71)	2.4 (0.25)^*^
Depth of inflammation	2.0 (0.00)	2.0 (0.00)	2.0 (0.00)	1.4 (0.55)	2.6 (0.52)^*^	3.6 (0.42)^*^	3.6 (0.23)	3.8 (0.48)	4.0 (0.60)
Neovascularization	1.2 (0.45)	1.0 (0.30)	1.0 (0.18)	2.0 (0.40)	2.0 (0.36)	1.2 (0.45)	2.0 (0.30)	2.0 (0.47)	1.6 (0.51)
Cellular repopulation	1.2 (0.45)	1.0 (0.22)	1.0 (0.19)	2.4 (0.36)	2.2 (0.48)	1.2 (0.17)^*^	4.0 (0.30)	4.0 (0.42)	3.4 (0.55)
Foreign body giant cells	0.2 (0.49)	0.4 (0.35)	0.4 (0.38)	0.2 (0.46)	0.2 (0.48)	0.4 (0.57)	1.0 (0.21)	1.2 (0.43)	2.6 (0.24)^*^

* *P < 0.05 vs. saline control group*.

In the inoculum groups of 10^6^ CFU/ml *E. coli* and 10^8^ CFU/ml *E. coli*, the scores of depth of inflammation, cellular repopulation and foreign body giant cells were significantly higher in the ThormalGEN mesh (*P* < 0.05). There was no neovascularization or cellular repopulation in polytetrafluoroethylene mesh (Table 4).

Compared with the contrast group, the reaction of inflammation was increased with the rise of the concentration of bacteria, except for the ThormalGEN mesh inoculated with 10^6^ CFU/ml *E. coli* (*P* < 0.05) (Table 4). On the other hand, no significant difference in neovascularization was observed with the increase of bacterial concentration (*P* > 0.05). Compared with the contrast group, the ThormalGEN mesh showed better tolerence of 10^6^ CFU/ml *E. coli* with respect to inflammation, depth of inflammation, neovascularization, cellular repopulation and foreign body giant cells (Table 4).

## Discussion

The use of prosthetic or biologic mesh in a contaminated or infected surgical site remains a controversy due to its postoperative complications such as infection. It is known that the different categories and concentrations of bacteria were important factors for the infection. Klinge et al. reported that the threshold concentration of *Staphylococcus aureus* leading to infection decreased to 10^2^-10^4^ CFU/ml after the use of artificial material (28). Merritt et al. also indicated that <10^6^ CFU/ml concentration of *Staphylococcus aureus* could cause infection of the implant (29). However, with respect to *E. coli*, the threshold concentration leading to infection has not been reported yet. In our study, we found that the concentrations of *E. coli* inoculated were positively correlated with the positive culture rate. The threshold concentration of *E. coli* for positive culture in polytetrafluoroethylene mesh, polypropylene mesh and ThormalGEN mesh was 10^2^ CFU/ml, 10^4^ CFU/ml and 10^4^ CFU/ml, respectively. However, when the concentration of *E. coli* was 10^2^ CFU/ml or 10^4^ CFU/ml, the positive culture rate among explanted mesh did not reach statistical significance (*P* > 0.05). On the contrary, it was statistical significant (*P* < 0.05) when the concentration of *E. coli* reached 10^6^ CFU/ml or 10^8^ CFU/ml. These data suggests that the bovine pericardium mesh materials may be more resistant to *E. coli* colonization than polytetrafluoroethylene mesh or polypropylene mesh when the concentration of *E. coli* is high. The mesh infection rate was the highest in polytetrafluoroethylene mesh and the lowest in the bovine pericardium mesh. The results of the wound infection rates in rats also supported this point. Thus, the bovine pericardium mesh is more resistant to infection in high concentration of *E. coli*, although the infection rate is still high, which was consistent with previous reports (4, 30).

The mesh resistance to *E. coli* is related to the material type and structure. Due to the multi-micropore structure of polytetrafluoroethylene mesh, macrophages could not enter the mesh and bacteria can easily hide within the mesh. In addition, the fibrous tissue is not able to grow into the mesh in short time, so the resistance to infection is relatively poor. We confirmed by histopathologic results that neutrophils were gathered on the surface of polytetrafluoroethylene mesh, not being able to enter the deep part of mesh on the 8th day after surgery. Furthermore, the fact that no new vessels or fibroblasts growing into the polytetrafluoroethylene mesh also decreased the resistance to infection. But for polypropylene mesh, the pore is larger than that of polytetrafluoroethylene mesh, allowing neutrophils to grow deeper into the mesh to remove the bacteria. The larger pores of polypropylene mesh were also helpful for the growth of new vessels and fibroblasts. As demonstrated by Pascual et al., that the larger the pore of mesh, the easier growing of the tissue into the mesh, and the better compatibility of the mesh (31). Cole et al. also reported that the bacterial clearance rate of large-pore lightweight polypropylene mesh was higher than that of biological mesh (32). Our histopathological results indicated that the number of neutrophils and foreign-body giant cells in the bovine pericardium mesh was the highest among the three kinds of meshes, which was easy to form abscess during infection. Cole et al. also reported that compared with lightweight polypropylene mesh, biological mesh was easier to form abscess during infection (32). However, the specific mechanism that why the biologic mesh had better *E. coli* resistance compared to polytetrafluoroethylene mesh and polypropylene mesh when the *E. coli* concentration is high remains further study. Thus, the efficacy of biological mesh still needs a long-term evaluation.

According to the European Hernia Society guidelines, there is no need for antibiotic prophylaxis in elective hernia repair for low-risk patients. For high risk patients such as recurrent hernia, old age, immunosuppressive condition, and long operating duration, antibiotic prophylaxis should be considered (33). Sanabria et al. reported that prophylactically use of antibiotics could reduce the wound infection rate by around 50% (34). Our study demonstrated that prophylactically use of ceftriaxone can help reduce the colonization rate, although it did not reach statistical significance. It is not difficult to comprehend that antibiotic prophylaxis was only useful when the contamination was not particularly severe. However, when the contamination was very serious, antibiotic prophylaxis alone was not able to cure the infection. Other measures such as surgical intervention and abscess drainage were needed to cure the infection. Our clinical experience indicates that bacterial migration might occur in incarcerated hernia surgery due to organ incarceration, especially the incarcerated bowel. When the bowel necrosis or perforation occurs, there is direct bacteria contamination in wound, so antibiotic prophylaxis is necessary. However, antibiotics might be useful in only a certain concentration of bacterial contamination and aseptic manipulation needs to be guaranteed.

Limitations of this study exist. Firstly, the results of this study were obtained from rat models. There might be bias when generalizing to human population. Secondly, the conclusion was based on the bovine pericardium mesh. Further studies of other biologic meshes derived from human tissues and porcine tissues are needed to systemically compare the biologic mesh with synthetic mesh.

In conclusion, with respect to *E. coli* resistance, the bovine pericardium mesh outperformed polytetrafluoroethylene mesh and polypropylene mesh when the *E. coli* concentration is higher than 10^6^ CFU/ml. Antibiotic prophylaxis is only useful when the contamination was not particularly severe. However, when the contamination is very serious, antibiotic prophylaxis alone is not able to cure the infection. Other measures such as surgical intervention and abscess drainage are needed to cure the infection. Polytetrafluoroethylene mesh is not recommended in contaminated hernia.

## Data Availability Statement

The original contributions presented in the study are included in the article/supplementary material, further inquiries can be directed to the corresponding author/s.

## Ethics Statement

The animal study was reviewed and approved by ethics committee of Renji Hospital, School of Medicine, Shanghai Jiao Tong University.

## Author Contributions

LY designed and coordinated the study. MZ, XL, and TC performed the experiments, acquired, and analyzed data. XX interpreted the data and wrote the manuscript. All authors approved the final version of the article.

## Conflict of Interest

The authors declare that the research was conducted in the absence of any commercial or financial relationships that could be construed as a potential conflict of interest.
